# Surgical Treatment Outcomes of Anterior‐Only Correction and Reconstruction for Severe Cervical Kyphotic Deformity with Neurofibromatosis‐1: A Retrospective Study with a 5‐Year Follow‐Up

**DOI:** 10.1111/os.14096

**Published:** 2024-05-20

**Authors:** Qiujiang Li, Liang Wang, Huiliang Yang, Xi Yang, Limin Liu, Lei Wang, Yueming Song

**Affiliations:** ^1^ Department of Orthopedic Surgery West China Hospital, Sichuan University Chengdu China

**Keywords:** Anterior‐only approach, Cervical kyphotic, Fixed, Neurofibromatosis

## Abstract

**Objectives:**

Currently, anterior‐only (AO), posterior‐only, and combined anterior–posterior spinal fusions are common strategies for treating cervical kyphosis in patients with neurofibromatosis‐1 NF‐1. Nevertheless, the choice of surgical strategy remains a topic of controversy. The aim of our study is to evaluate the safety and effectiveness of anterior decompression and spinal reconstruction for the treatment of cervical kyphosis in patients with NF‐1.

**Methods:**

Twelve patients with NF‐1‐associated cervical kyphotic deformity were reviewed retrospectively between January 2010 and April 2020. All patients underwent AO correction and reconstruction. The X‐ray was followed up in all these patients to assess the preoperative and postoperative local kyphosis angle (LKA), the global kyphosis angle (GKA), the sagittal vertical axis, and the T1 slope. The visual analog scale score, Japanese Orthopedic Association (JOA) score, and neck disability index (NDI) score were used to evaluate the improvement inclinical symptoms. The results of the difference in improvement from preoperatively to the final follow‐up assessment were assessed using a paired *t*‐test or Mann–Whitney *U*‐test.

**Results:**

The LKA and GKA decreased from the preoperative average of 64.42 (range, 38–86) and 35.50 (range, 10–81) to an average of 16.83 (range, −2 to 46) and 4.25 (range, −22 to 39) postoperatively, respectively. The average correction rates of the LKA and GKA were 76.11% and 111.97%, respectively. All patients had achieved satisfactory relief of neurological symptoms (*p* < 0.01). JOA scores were improved from 10.42 (range, 8–16) preoperatively to 15.25 (range, 11–18) at final follow‐up (*p* < 0.01). NDI scores were decreased from an average of 23.25 (range, 16–34) preoperatively to an average of 7.08 (range, 3–15) at the final follow‐up (*p* < 0.01).

**Conclusion:**

Anterior‐only correction and reconstruction is a safe and effective method for correcting cervical kyphosis in NF‐1 patients. In fixed cervical kyphosis cases, preoperative skull traction should also be considered.

## Graphical abstract

Our data suggest that anterior‐only (AO) correction and reconstruction is a safe and effective method for correcting cervical kyphosis in NF‐1 patients. In fixed cervical kyphosis cases, preoperative skull traction should also be considered.

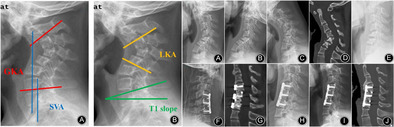

## Introduction

Neurofibromatosis‐1 (NF‐1) is a genetic disease caused by mutations in the NF‐1 tumor suppressor gene. It is inherited in an autosomal dominant manner and has an incidence of 1 in every 3000 individuals.^1^, ^2^ Spinal deformity is the most common musculoskeletal manifestation of NF‐1 and is divided into dystrophic and non‐dystrophic types. Dystrophic spinal deformities are associated with neurofibromas, characterized by direct influence of neurofibromas on the structure of the spine, resulting in bone destruction and spinal instability. This type of spinal deformity is often accompanied by localized or extensive bone loss. Non‐dystrophic types might involve other factors, such as local compression of the tumor or an indirect link between neurofibromas and skeletal development. In this case, the structural changes in the spine are mainly due to uneven growth of the spine rather than direct destructive effects of the neurofibroma. Although NF‐1‐associated dystrophic cervical kyphosis is relatively rare in clinical practice, it progresses rapidly and, if allowed to progress naturally without treatment, might lead to further progression of the deformity and severe neurological deficits, including spinal cord injury and life‐threatening paralysis.^3^, ^4^, ^5^ Calvert et al.^6^ conducted a review of the natural history of 32 patients with neurofibromatosis‐associated dystrophic spinal deformities over an average period of 3.6 years. They found that initial scoliosis increased at an average rate of 8.1 per year. Therefore, early diagnosis and treatment of neurofibromatosis cervical kyphosis deformity are crucial.

Currently, anterior‐only (AO), posterior‐only (PO), and combined anterior–posterior (AP) spinal fusions are common strategies for treating cervical kyphosis in patients with NF‐1.^7^, ^8^, ^9^, ^10^, ^11^ Nevertheless, the choice of surgical strategy remains a topic of controversy. AP approach has been shown to provide a more comprehensive correction for cervical kyphosis and sagittal balance in patients with NF1.^8^ However, due to its higher invasiveness, longer operation time, and greater blood loss, the surgical risk for this method is correspondingly increased, so in practical clinical practice, the use of this method remains widely controversial. A single surgical approach (AO or PO) for the treatment of NF‐1‐induced severe cervical kyphosis not only simplifies the surgical procedure but also greatly reduces the risk of surgery‐related complications and, thus, the patient's recovery time. Stoker et al.^12^ used the PO method to treat NF‐1 cervical kyphosis in a 14‐year‐old child and found that PO spinal resection was a safe and effective treatment option. Although its corrective effect is credible, surgical intervention via a posterior approach and possible complications after surgery remain problems that cannot be ignored. Choksey et al.^13^ and Gu et al.^14^ reported cases of kyphosis treated with a single anterior decompression and fusion technique with high postoperative satisfaction, suggesting that the AO method has important advantages in avoiding nerve damage and reducing neurovascular structure damage caused by transverse mass screw fixation or threading. However, these studies reported short follow‐up periods and insufficient long‐term follow‐up data, especially to assess postoperative fusion, vertebral erosion, and new kyphosis. Therefore, the aim of our study, with a 5‐year follow‐up, is (i) to evaluate the safety and effectiveness of AO correction and reconstruction for the treatment of cervical kyphosis in patients with NF‐1 and (ii) to evaluate the effectiveness of skull traction combined with AO approach correction.

## Materials and Methods

### Patients

This study received approval from the Medical Ethics Committee of our hospital (No. 2023‐331). A retrospective analysis was conducted on patients with NF‐1‐related cervical kyphosis who underwent surgery at our institution from January 2010 to April 2020. The inclusion criteria consisted of the following: (i) diagnosis of NF‐1 according to the National Institutes of Health (1987) guidelines;^15^ (ii) Cobb angle exceeding 40°; (iii) patients who underwent the AO approach; and (iv) patients with rapid progression of the deformity and severe neurologic compromise. Exclusion criteria: (i) kyphosis of the cervical spine caused by infection, trauma, degeneration, or idiopathic factors; (ii) other types of neurofibromatosis, non‐dystrophic cervical kyphosis, and primary or secondary malignant tumors of the cervical spine; (iii) simple deformity of upper cervical spine or craniocervical junction; and (iv) a history of spinal surgery. The last 12 patients were included in the study, with a mean age of 24.67 years (range, 14–41 years). The mean follow‐up time was 56.33 months (range, 34–98 months).

### Traction Procedure

Skull traction was placed under local anesthesia in all patients with fixed cervical kyphosis. The patient was supine, and the traction was gradually adjusted from parallel to hyperextension by placing a blanket under the shoulders. The initial traction weight was 3 kg, and this process lasted for 1 week and gradually increased to 1/8 of the patient's body weight. X‐ray examination was performed weekly to observe whether the Cobb angle improved significantly. Traction was maintained until Cobb angle did not progress. During this period, neurological examinations were performed twice daily. If discomfort occurred, the traction weight was temporarily reduced.

### Surgical Procedures

Following successful anesthesia, the patient was positioned supine, and a right anterior transverse incision was made. This incision allowed for the gradual exposure of the prevertebral fascia layer by layer using the classic anterior Smith–Robinson approach. A corpectomy procedure was then performed on the dysplastic vertebral body. The Caspar spreader was utilized at multiple levels, along with intraoperative cranial traction, to enlarge the intervertebral space, release anterior structures, clear vertebral osteophytes, and restore cervical lordosis. To further correct the kyphosis, a titanium mesh cage or PEEK cage filled with autogenous bone graft was inserted. Additionally, a plate screw system of appropriate length was selected for fixation. Finally, the wound was closed using a gradual suturing technique. Postoperation, a drainage tube was routinely placed and removed 24–72 h later. Subsequently, a neck brace was worn for ambulation.

### Radiographic Assessment

**Figure 1:**
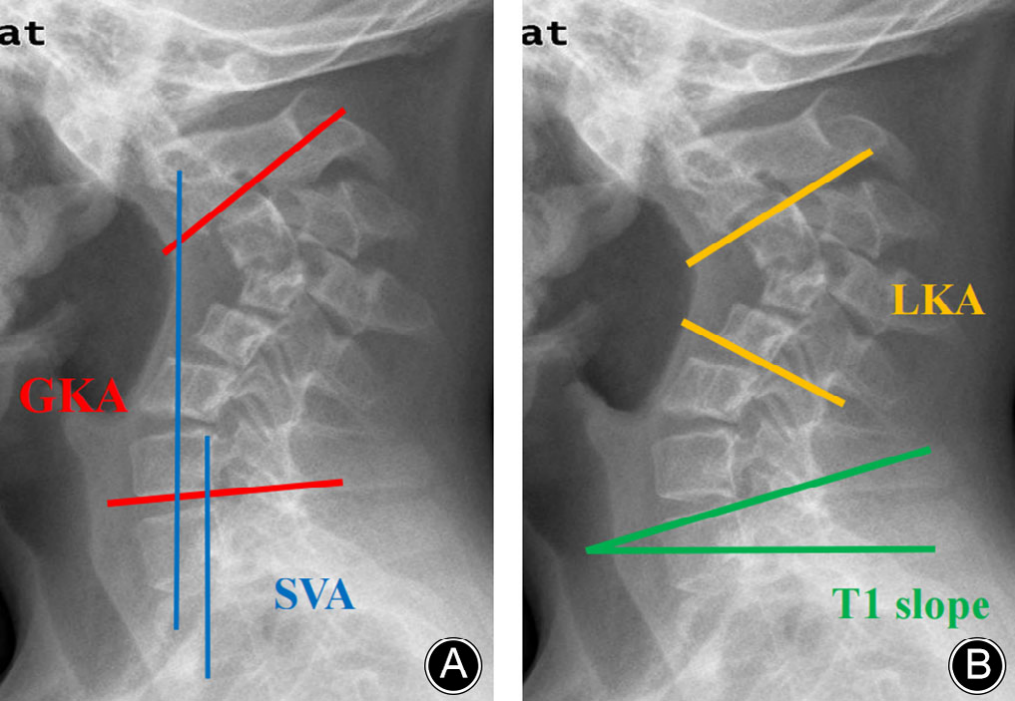
Radiological parameters measurement methods. Local kyphosis angle (LKA): the angle formed by the intersection of extended lines along the posterior borders of the upper and lower vertebral bodies in the kyphotic segment. Global kyphosis angle (GKA): the angle formed between C2 and C7 lower endplate. Positive values were assigned to the lordotic curvature and negative values to the kyphotic curvature. Sagittal vertical axis (SVA): the distance between the posterior end of the end plate on the C7 vertebra and the vertical line from the center of the C2 body. T1 slope: the angle between the extension of the T1 upper endplate and the horizontal line.

All the patients underwent routine preoperative examination, including static and lateral flexion/extension X‐ray, computed tomography (CT) scan, and magnetic resonance imaging (MRI). Some radiological parameters (local kyphosis angle, global kyphosis angle, sagittal vertical axis, and T1 slope) were measured preoperatively, 1 week postoperatively, and at the final follow‐up. The vertebra with the maximum degree of inclination at cephalad and caudal is defined as the upper and lower vertebral bodies of the kyphotic segment. The local kyphosis angle (LKA) is the angle formed by the intersection of extended lines along the posterior borders of the upper and lower vertebral bodies in the kyphotic segment. The global kyphosis angle (GKA) is the angle formed between C2 and C7 lower endplate. Positive values were assigned to the lordotic curvature and negative values to the kyphotic curvature. The sagittal vertical axis (SVA) is the distance between the posterior end of the end plate on the C7 vertebra and the vertical line from the center of the C2 body. The T1 slope is the angle between the extension of the T1 upper endplate and the horizontal line. The correction rate of the Cobb angle was calculated as follows: (postoperative Cobb angle − preoperative Cobb angle)/preoperative Cobb angle × 100%. All imaging parameters were measured by two surgeons not involved in the initial procedure and analyzed using the mean of the two measurements (Figures 1).

### Interobserver and Intraobserver Agreement

We randomly selected all patients to test the interobserver and intraobserver agreement. First, two spine surgeons who did not participate in the operation independently measured the radiographic parameters with no knowledge of the clinicopathological data. Two weeks later, these same spine surgeons performed a second independent measurement of the radiographic parameters. The intraclass correlation coefficient (ICC) was used to assess the interobserver and intraobserver reliability of quantitative radiographic parameters, and ICC ≥0.75 was considered to have good reliability.

### Clinical Evaluation

The demographic characteristics collected included age, gender, postoperative complications, and duration of follow‐up. Postoperative complications included cerebrospinal fluid leakage, postoperative hematoma, dysphagia, hoarseness, C5 nerve root paralysis, and pseudarthrosis. The Japanese Orthopedic Association (JOA) score was used to assess neurological status; the neck disability index (NDI) score was used to assess health related quality of life; and the visual analog scale (VAS) score was used to assess neck pain preoperatively, postoperatively, and at final follow‐up. All patients’ follow‐up information was collected during outpatient visits or telephone follow‐up.

### Statistical Analysis

Continuous variables with a normal distribution are reported as mean ± standard deviation, while non‐normally distributed variables are presented as median and interquartile range (IQR). A *t*‐test was used for normal data, and the Mann–Whitney *U*‐test was used for non‐normal data. All statistical analyses were performed using SPSS 26.0 (SPSS, Chicago, IL), and *p*‐values <0.05 were considered statistically significant.

## Results

### Demographic Data and Clinical Characteristics

The study included 12 consecutive patients, 7 male and 5 female, with a mean age of 24.67 years (range, 14–41 years) at the time of surgery. All patients had dysplasia, including 7 cases of fixed cervical kyphosis and 5 cases of flexible cervical kyphosis. In this study, 7 cases of fixed cervical kyphosis were treated with cervical skull traction, the traction weight was maintained at 6–10 kg, and the average follow‐up time was 56.33 months (range, 34–98 months) (Table 1).

**Table 1:** Demographic and surgical data of patients

Patient number	Age (years)	Gender	Vertebral dysplasia	Flexibility	Traction (Days, Kg)	Surgery procedures	Follow‐up (months)
1	15	Male	Yes	Fixed	21, 10	C2‐6ACDF	36
2	16	Male	Yes	Fixed	10, 8	C3‐5ACDF	98
3	18	Female	Yes	Fixed	12, 8	C3‐4ACDF + C5ACCF	87
4	36	Female	Yes	Flexible	‐	C2‐5ACDF	56
5	24	Male	Yes	Flexible	‐	C3‐5ACDF	34
6	14	Male	Yes	Fixed	14, 8	C3‐6ACDF	49
7	19	Male	Yes	Fixed	21, 10	C4‐5ACDF + C6ACCF	58
8	31	Female	Yes	Flexible	‐	C2‐5ACDF	61
9	19	Female	Yes	Fixed	7, 6	C2‐5ACDF	44
10	41	Female	Yes	Flexible	‐	C3‐6ACDF	54
11	35	Male	Yes	Flexible	‐	C4‐6ACDF	39
12	28	Male	Yes	Fixed	14, 8	C3‐5ACDF + C6ACCF	60

### Preoperative and Postoperative Local Kyphosis Angle and Global Kyphosis Angle

**Figure 2:**
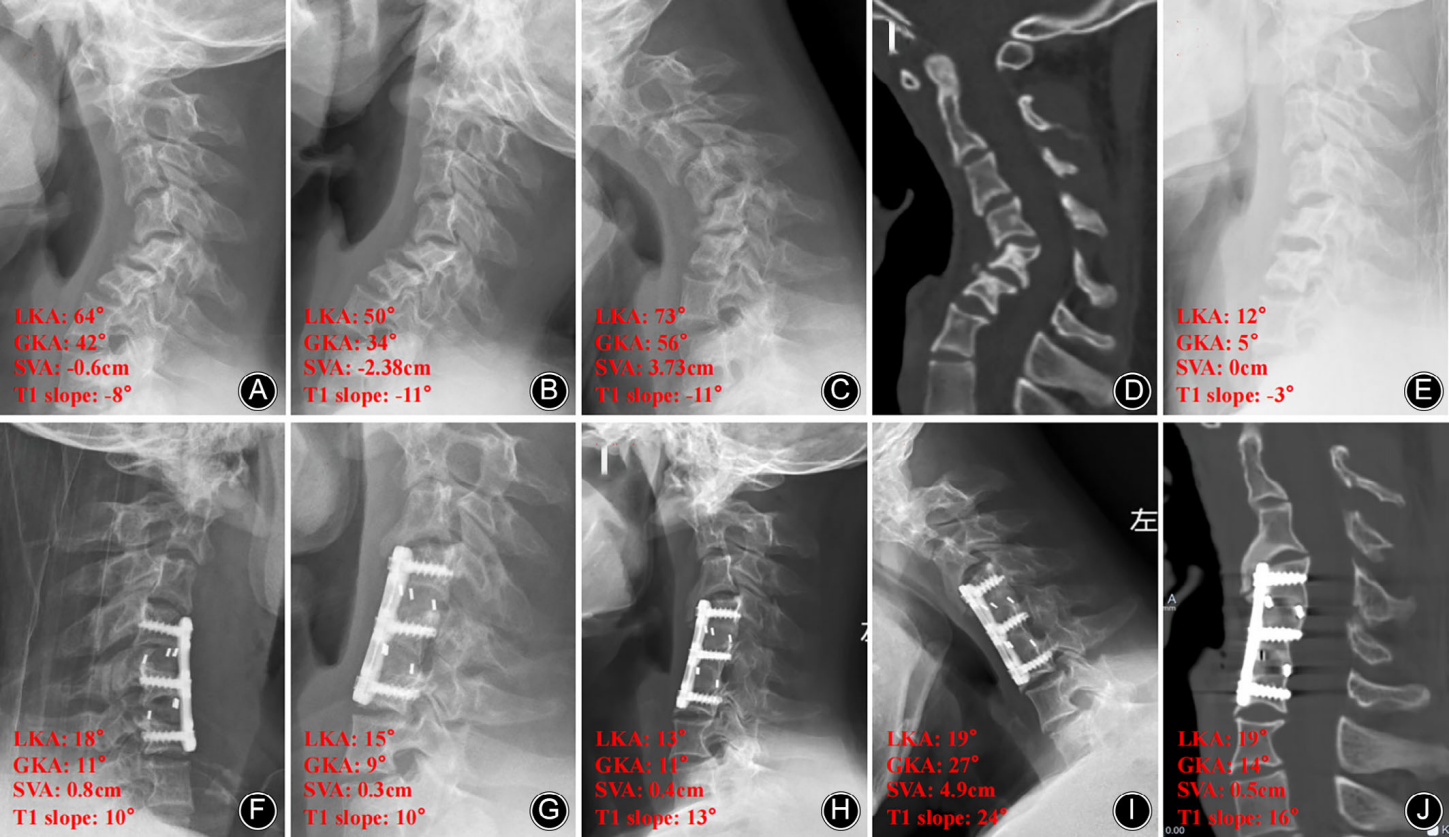
Case 7. A 16‐year‐old boy with NF‐1 cervical kyphosis. Preoperative lateral X‐ray (A) and computed tomography (CT) scan (D) showing significant scallop of C5 and C6 vertebrae. The local kyphosis angle (LKA) and global kyphosis angle (GKA) were 64° and 42°, respectively. Preoperative flexion–extension X‐ray (B, C) showing fixed cervical kyphosis. The LKA and GKA were 50° and 34°, respectively, in extension X‐ray, and 73° and 56°, respectively in flexion X‐ray (C). Post‐traction X‐ray (E) showed that the LKA and GKA were 12° and 5° after 10 days of continuous skull traction. Postoperative X‐ray (F) showed that the LKA and GKA were 18°and 11°after C4–6 anterior cervical corpectomy and fusion. Kyphosis correction was well maintained based on postoperative 3 months X‐ray (F), 8 months X‐ray (G), 57 months flexion X‐ray (H), and 57 months extension X‐ray (I), without instrumentation failure (J). At 57‐month follow‐up, the postoperative LKA and GKA reduced to 19° and 14°, respectively.

**Figure 3:**
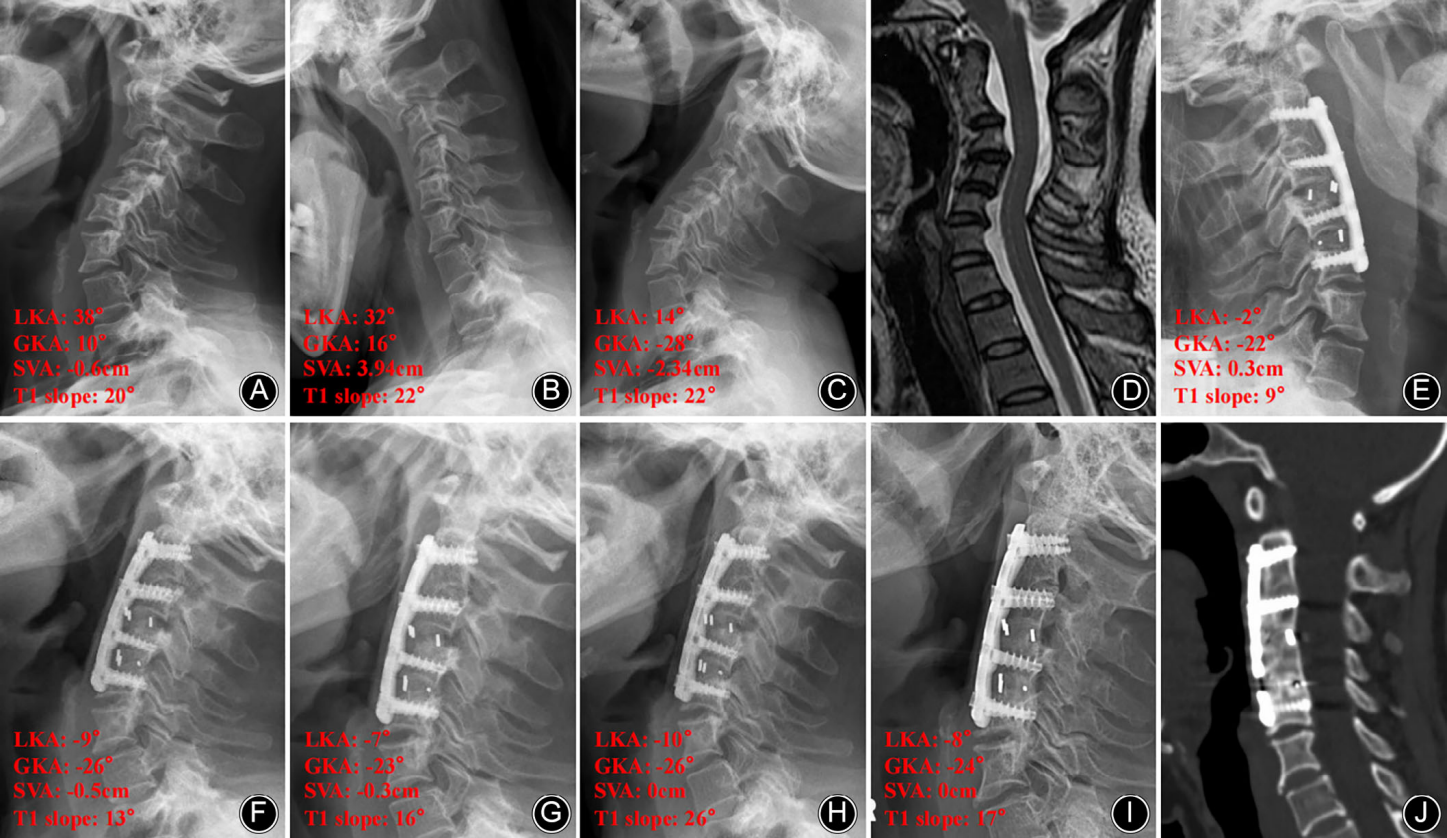
Case 4. A 36‐year‐old woman with Neurofibromatosis‐1 (NF‐1) cervical kyphosis. Preoperative lateral X‐ray (A) showing significant dislocation and scallop of C3 and C4 vertebrae. The local kyphosis angle (LKA) and global kyphosis angle (GKA) were 38° and 10°, respectively. Preoperative flexion–extension X‐ray (B, C) showing flexible cervical kyphosis. The LKA and GKA were 14° and −28°, respectively, in extension X‐ray (C). Preoperative magnetic resonance imaging (MRI) (D) shows compression of the spinal cord at C3/4 level. Postoperative X‐ray (E) showed that the LKA and GKA were −2° and −22° after C3–6 anterior cervical corpectomy and fusion. Kyphosis correction was well maintained at postoperative 12‐month X‐ray (F), 24‐month X‐ray (G), 54‐month X‐ray (H), and 96‐month X‐ray (I), without instrumentation failure (J). At 96‐month follow‐up, the postoperative LKA and GKA reduced to −8° and −24°, respectively (I, J).

Intraobserver and intraobserver agreement between two spine surgeons was near perfect; the ICC of all variables was >0.75, defined as excellent reliability. The LKA and GKA decreased from the preoperative average of 64.42 (range, 38–86) and 35.50 (range, 10–81) to an average of 16.83 (range, −2 to 46) and 4.25 (range, −22 to 39) postoperatively, respectively (both *p* < 0.01). The average correction rates of the LKA and GKA were 76.11% and 111.97%, respectively. At the final follow‐up, the average LKA and GKA were 19.58 (range, −8 to 49) and 8.08 (range, −24 to 39), respectively（typical cases Figures 2 and 3） (Table 2).

**Table 2:** Preoperative and Postoperative LKA and GKA

Number	LKA					GKA				
	Preoperative	Postoperative	Final follow‐up	Correction degree	Correction rate	Preoperative	Postoperative	Final follow‐up	Correction degree	Correction rate
1	75	7	6	68	90.67%	43	6	6	37	86.05%
2	86	46	49	40	46.51%	81	39	39	42	51.85%
3	56	10	12	46	82.14%	34	4	10	30	88.24%
4	38	‐2	−8	40	105.26%	10	−22	−24	32	320.00%
5	73	19	28	54	73.97%	45	10	25	35	77.78%
6	49	11	18	38	77.55%	19	−17	−15	36	189.47%
7	64	18	19	46	71.88%	42	11	14	31	73.81%
8	76	16	23	60	78.95%	51	8	15	43	84.31%
9	58	10	13	48	82.76%	24	−6	2	30	125.00%
10	69	25	26	44	63.77%	32	11	13	21	65.63%
11	52	9	13	43	82.69%	16	−2	−3	18	112.50%
12	77	33	36	44	57.14%	29	9	15	20	68.97%
Mean (IQR/SD)	64.42 (53.00, 75.75)	16.83 (9.25, 23.50)	19.58 (12.25, 27.50)	47.58 (40.75, 52.50)	76.11 (66.00, 83.00)%	35.50 (20.25, 44.50)	4.25 ± 15.54	8.08 (−1.75, 15.00)	31.2 (23.25, 36.75)	111.97 ± 74.89%
U		−4.100	−4.072				−3.610	−3.263		
* *p*‐value		<0.001	<0.001				<0.001	0.001		

* Compared with preoperatively.

### Local Kyphosis Angle and Global Kyphosis Angle of Preoperative and Post‐Traction

Among 12 patients, 7 patients with fixed cervical kyphosis underwent cervical skull traction. The LKA and GKA decreased from the preoperative average of 66.43 (range, 49–86) and 38.86 (range, 19–81) to an average of 31.14 (range, 12–56) and 16.86 (range, 3–47) postoperatively, respectively (both *p* < 0.05). The average correction rates of the LKA and GKA after traction were 54.80% and 60.45%, respectively (Table 3).

**Table 3:** LKA and GKA of preoperative and post‐traction

Number	LKA				GKA			
	Preoperative	Post‐traction	Correction degree	Correction rate	Preoperative	Post‐traction	Correction degree	Correction rate
1	75	44	31	41.33%	43	22	21	48.84%
2	86	56	30	34.88%	81	47	34	41.98%
3	56	24	32	57.14%	34	17	17	50.00%
6	49	21	28	57.14%	19	3	16	84.21%
7	64	12	52	81.25%	42	5	37	88.10%
9	58	21	37	63.79%	24	10	14	58.33%
12	77	40	37	48.05%	29	14	15	51.72%
Mean (IQR)	66.43 (56.00, 77.00)	31.14 (21.00, 44.00)	35.29 (30.00, 37.00)	54.80 (41.00, 64.00)%	38.86 (24.00, 43.00)	16.86 (5.00, 22.00)	22.00 (15.00, 34.00)	60.45 (49.00, 84.00)%
U		−2.945				−2.236		
* *p*‐value		0.003				0.025		

* Compared with preoperatively.

### Preoperative and Postoperative Sagittal Vertical Axis and T1 Slope

The SVA increased from the preoperative average of −0.48 cm (range, −2.4 to 2.9 cm) to an average of 0.90 cm (range, −0.4 to 2.1 cm) after surgery and 0.67 cm (range, −1.6 to 1.8 cm) at the final follow‐up. The average T1 slope was −4.25 (range, −24 to 19) before surgery, 2.67 (range, −19 to 14) after surgery, and 1.25 (range, −17 to 14) at the final follow‐up (Table 4).

**Table 4:** Preoperative and Postoperative SVA and T1 slope

Number	SVA			T1 slope		
	Preoperative	Postoperative	Final follow‐up	Preoperative	Postoperative	Final follow‐up
1	0.3	1.9	1.8	11	9	10
2	−2.1	1.1	−1.6	−24	−19	−16
3	−2.4	1.6	1.4	19	11	14
4	−2.3	0.3	0	−20	−9	−17
5	1.4	−0.4	0.9	7	1	5
6	2.9	0.5	0.9	6	14	11
7	−1.1	0	0.4	−8	−3	−13
8	−0.9	1.9	1.4	13	4	14
9	1.7	0.3	0.1	−10	8	5
10	−1.6	1.1	1.2	−9	8	3
11	1.1	0.4	0.1	−17	4	−3
12	−2.8	2.1	1.4	−19	4	2
Mean (IQR)	−0.48 (−2.25, 1.33)	0.90 (0.30, 1.83)	0.67 (0.10, 1.40)	−4.25 (−18.50, 10.00)	2.67 (−2.00, 8.75)	1.25 (−10.50, 10.75)
U		1.909	1.505		1.070	0.867
* *p* value		0.056	0.132		0.285	0.386

* Compared with preoperatively.

### Clinical Measurement and Functional Outcomes

All patients had achieved satisfactory relief of neurological symptoms. JOA scores were improved from 10.42 (range, 8–16) preoperatively to 15.25 (range, 11–18) at final follow‐up (*p* < 0.01). NDI scores were decreased from an average of 23.25 (range, 16–34) preoperatively to an average of 7.08 (range, 3–15) at the final follow‐up (*p* < 0.01). Patients had achieved effective pain relief at the final follow‐up with an average of 1.42 (*p* < 0.01) (Table 5).

**Table 5:** Clinical measurement and functional outcomes

Number	VAS		JOA		NDI	
	Preoperative	Final follow‐up	Preoperative	Final follow‐up	Preoperative	Final follow‐up
1	5	1	11	18	21	4
2	7	2	8	14	17	3
3	4	1	16	18	19	4
4	6	0	12	15	19	8
5	8	1	9	11	34	8
6	5	0	10	18	17	6
7	8	3	9	12	31	7
8	7	2	12	18	23	7
9	7	2	10	16	35	11
10	5	0	12	18	18	8
11	8	3	8	11	29	15
12	6	2	8	14	16	4
Mean (IQR/SD)	6.33 (5.00, 7.75)	1.42 (0.25, 2.00)	10.42 (8.25, 12.00)	15.25 ± 2.83	23.25 (17.25, 30.50)	7.08 (4.00, 8.00)
U		−4.186		3.321		−4.167
* *p*‐value		<0.001		0.001		<0.001

* Compared with preoperatively.

### Complications

None of the patients had neurological complications or surgical site infections. At final follow‐up, all 12 patients had solid bone fusion. There was no case of internal fixation failure.

## Discussion

Spinal deformity is one of the most common complications of NF‐1. Although the incidence of cervical spine is relatively low, often cervical kyphosis is the main deformity, once the disease progresses rapidly, the symptoms are severe, and the treatment is difficult. In a retrospective study of 33 patients with severe cervical deformity secondary to neurofibromatosis, six patients developed mild paralysis, six developed respiratory distress, and all underwent surgery with solid healing.^16^ Therefore, early surgical intervention can effectively reduce the potential risk of paralysis due to the development of cervical kyphosis and the failure of brace treatment. Therefore, we completed a 5‐year follow‐up study of AO correction and reconstruction for NF‐1 patients. The results showed that AO correction and reconstruction is a safe and effective method for correcting cervical kyphosis in NF‐1 patients. In fixed cervical kyphosis cases, preoperative skull traction should also be considered.

### Surgical Approachs

Cervical kyphosis secondary to NF‐1 is different from the common cervical kyphosis, including the severity and flexibility of cervical kyphosis, vertebral body deformation, bone quality, and intraspinal abnormalities.^7^, ^17^, ^18^ The potential risk of spinal cord injury is often high during correction, and even the transient pursuit of more ideal correction might lead to severe spinal cord injury and ischemia. Therefore, the choice of surgical strategy for NF‐1 cervical kyphosis remains controversial. Helenius et al.^3^ reviewed 22 children with NF‐1 cervical kyphosis and compared the clinical results of the AP approach with those of the PO approach and found that the AP approach provided better correction of cervical kyphosis than the PO approach. Ma et al.^5^ reported on 8 patients and concluded that a more aggressive AP approach is the most reliable surgical strategy for patients with kyphosis angles greater than 50° and poor flexibility. Lin et al.^8^ compared the results of the three approaches and found that the AP approach resulted in optimal correction of cervical kyphosis and sagittal balance and greater patient satisfaction in patients with NF1. Stoker et al.^12^ reported a PO approach for the treatment of NF‐1 cervical kyphosis in a 14‐year‐old child and found that PO vertebral column resection represents a safe and efficacious treatment. Although the correction rate was reliable, surgical invasion and postoperative complications of the posterior approach should not be ignored. Choksey et al.^13^ reported a patient with satisfactory postoperative results after correction of cervical kyphosis with a single anterior decompression and fusion procedure, suggesting that the AO approach did not damage the nerves and avoided the risk of damage to important neurovascular structures caused by pedicle or lateral mass screw fixation. Gu et al.,^14^ in a short‐term follow‐up of 7 patients who underwent the AO approach, for NF‐1‐related cervical kyphosis, found that AO correction and reconstruction was an alternative option for the treatment of NF‐1‐associated severe cervical kyphosis when deformity was localized, flexible, or fixed. In our study, the average correction rates of the LKA and GKA were 76.11% and 111.97%, respectively, which is a satisfying result compared to that reported in previous studies (Tables S1 and S2). The AO approach also provided more reliable and better correction of cervical kyphosis without further progress. Although the anterior approach has significant advantages, indications should be carefully considered. The choice of surgical approach is influenced by factors such as the degree of kyphosis, the extent of vertebral deformation, the flexibility of kyphosis curve, the density of vertebrae, the specific location of nerve compression, and the presence of intraspinal or paravertebral neurofibromas. For patients with>5 fusion levels, the AP approach is effective in preventing junctional kyphosis. The AO approach is used when there are concerns about curve stiffness, size, nerve compression, and poor bone condition of the patient. Ma et al.^5^ reported on 8 patients and concluded that a more aggressive AP approach was the most reliable surgical strategy for patients with kyphosis angle greater than 50 and less flexibility. For severe kyphosis, a posterior–anterior surgical approach was chosen, allowing for load‐bearing reconstruction, because normally, a vertebral body supports just 36% of the axial load.

### Preoperative Cervical Traction

Preoperative continuous cervical traction could correct cervical kyphosis to a certain extent, release the muscles around the cervical spine and facet joints, provide a wider operating space for the anterior approach, and reduce the surgical risk and difficulty.^19^, ^20^, ^21^ In cases of NF‐1‐associated kyphosis, less traction is usually indicated due to the fragility of the dystrophic cervical spine.^22^ In our study, 7 cases of fixed cervical kyphosis were treated with cervical skull traction, and the traction weight was maintained at 6–10 kg for a total duration of 1–3 weeks. The LKA and GKA decreased from the preoperative average of 66.43 and 38.86 to an average of 31.14 and 16.86 postoperatively, respectively. The average correction rates of the LKA and GKA after traction were 54.80% and 60.45%, respectively. Previous studies have shown that an anterior strategy should be used when the deformity is flexible or fixed without facet ankylosis.^17^ Han et al.^23^ stated that spinal cord flexibility evaluated by traction and anterior compression should be the basis of the AO approach. Thus, for patients with fixed cervical kyphosis, release by preoperative skull traction for 1–3 weeks has been shown to increase flexibility and partially reduce kyphosis. After a single anterior cervical fusion reconstruction, the correction rate of all patients was good, which proved that continuous cervical traction before operation was safe. Based on this, we recommend reassessing the patient's neck flexibility and recovery potential before and after surgery, while carefully considering the choice of surgical approach and the indications for AO.

### Adequate Correction

Adequate postoperative correction of the kyphotic deformity can result in stable spinal reconstruction while also improving the patient's appearance.^24^, ^25^ Posterior correction of cervical kyphosis is limited by vertebral dysplasia and weak screw extraction resistance.^26^ The anterior approach restores sufficient cervical flexibility by removing the intervertebral disc, facet joints, and contracture of soft tissue, so that a relatively normal cervical lordotic curvature can be achieved with appropriate corrective force. There is a potentially high risk of spinal cord injury during correction, and therefore intraoperative neurophysiological monitoring is critical for achieving effective and adequate correction. Adequate correction, while allowing for more stable spinal reconstruction, also increases the risk of iatrogenic nerve injury to some extent. Relevant studies have also suggested that a correction rate of approximately 80% should be an adequate and safe degree of correction.^3^, ^13^, ^27^ In our study, the average correction rate of the LKA was 76.11%, and there was no further progression at long‐term follow‐up. However, careful long‐term follow‐up should be observed in all cases for signs of progressive vertebral erosion. Skeletal dystrophy is a persistent pathological change, and the follow‐up time of some studies is short, so there are few signs of vertebral erosion reported. Helenius et al.^3^ reported vertebral erosion in all patients at the 24‐month follow‐up, while Wang et al.^7^ reported significant vertebral erosion in 5 patients treated at the 33‐month follow‐up. No patients in this study experienced significant vertebral body erosion.

### BMD in NF‐1

Neurofibromatosis‐1‐associated systemic skeletal dystrophy presents with osteoporosis or osteopenia.^28^, ^29^ Studies have shown that early bone mineral density (BMD) loss can be as high as 50%.^30^, ^31^ NF‐1 patients requiring surgical intervention had a more pronounced decline in BMD. In this study, decreased bone mineral density was found in both X‐ray and CT examinations before the operation. Intraoperatively, cortical thinning and brittleness were confirmed. Although risedronate and oral vitamin D3 have been shown to be effective in improving bone mineral density in adults with NF‐1, there is no official consensus on preventive use in childhood.^32^ Therefore, to achieve the stability of spinal reconstruction, more anchors and sufficient and high‐quality bone grafts are very important.

### Postoperative PJK

Good fusion rates were achieved with different approaches, but postoperative fusion failure, pseudarthrosis, and loss of correction were also noted during follow‐up. Sirois and Drennan^33^ also reported that NF‐1 with the PO approach resulted in 72% and 53% correction failure in patients with cervical kyphosis. Junctional kyphosis is one of the most common postoperative complications of cervical deformity surgery and has been reported in several studies.^34^, ^35^ The probable cause of junctional kyphosis after surgery for NF‐1 cervical kyphotic deformity is related to vertebral body erosion and postoperative spinal imbalance. Lin et al.^8^ reported 81 patients with NF‐1 cervical kyphosis deformity, and 11 patients developed junctional kyphosis after operation. The AP approach combining more than five levels of fusion could effectively prevent junctional kyphosis compared with AO and PO approaches. However, in this study, no patients developed junctional kyphosis. Our experience is that spinal fusion at all dysplastic levels has been shown to be effective in reducing the risk of junctional kyphosis in the medium to long term.

### Strengths and Limitations

Currently, the choice of surgical strategy remains a topic of controversy. Our results showed that AO correction and reconstruction is a safe and effective method for correcting cervical kyphosis in NF‐1 patients. In fixed cervical kyphosis cases, preoperative skull traction should also be considered. However, there are still some limitations of our study. First, this was a retrospective study in a single center, and the small sample size was another limitation of the study. Further prospective large‐scale randomized controlled trials are needed to confirm the current findings. Second, there is a lack of clinical observation of the results of different approaches for the treatment of NF‐1 cervical kyphosis.

## Conclusion

The results of this study suggest that preoperative skull traction can effectively increase flexibility and partially reduce cervical kyphosis. Combined with a single anterior surgical resection of pathological structures and reconstruction to correct and maintain cervical kyphosis and achieve solid bone fusion, it is a safe and effective method for correcting cervical kyphosis in NF‐1 patients.

## Conflict of Interest Statement

The authors report no conflict of interest concerning the materials or methods used in this study or the findings specified in this paper.

## Ethics Statement

This study was performed in line with the principles of the Declaration of Helsinki. Approval was granted by the Ethics Committee of the West China hospital. Written informed consent was obtained from the parents.

## Author Contributions

Lei Wang and Yueming Song contributed to the study conception and design. Material preparation was performed by Xi Yang, Limin Liu, and Qiujiang Li. Data collection and analysis were performed by Qiujiang Li, Liang Wang, Huiliang Yang, and Limin Liu. The first draft of the manuscript was written by Qiujiang Li and Liang Wang, and all authors commented on previous versions of the manuscript. All authors read and approved the final manuscript.

## Funding Information

This study was supported by the Science and Technology Project of the Health Planning Committee of Sichuan (2022NSFSC1528, 2022YFS0016 and 2022YFS0260); the 1‐3‐5 project for disciplines of excellence, West China 14 Hospital, Sichuan University (ZYGD21001); and the National Natural Science Foundation of China (82072386 and 82102521).

## Supporting information


**Table S1.** Clinical results of in different literatures.
**Table S2.** Comparison of our 12 cases and 80 cases in literatures.

## Data Availability

The datasets used during the current study are available from the corresponding author on reasonable request.
