# Supplementary material for: Surgical Treatment Outcomes of Anterior‐Only Correction and Reconstruction for Severe Cervical Kyphotic Deformity with Neurofibromatosis‐1: A Retrospective Study with a 5‐Year Follow‐Up
Source: Orthop Surg. 2024 May 20;16(7):1631–41. doi: 10.1111/os.14096 (PMC11216833; doi:10.1111/os.14096)
Supplement: Supplementary file 1 — Table S1. Clinical results of in different literatures. Table S2. Comparison of our 12 cases and 80 cases in literatures. [file OS-16-1631-s001.docx]

| Authors | Case | Age, years | Preoperative Cobb | Postoperative Cobb | Traction | Surgical procedure | Correction rate(%) | Operation time, min | Blood loss, ml | Follow up |
| --- | --- | --- | --- | --- | --- | --- | --- | --- | --- | --- |
| Yonezawa 2003[10] | 1, M | 15 | 72 | 35 | - | A+P | 51.4 | - | - | 16m |
| Laohacharoensombat 2010[21] | 1, M | 8 | 120 | 55 | Skull traction | AO | 54 | - | - | 6y |
| Ma 2011[5] | 8(3M, 5F) | 19(12-38) | 58.5(45-78) | 2.5 | - | A+P | 95.7 | - | - | 21.1(6-36)m |
| Wu 2012[26] | 1M | 18 | 125 | 30 | Cervical suspensory traction | A+P | 76 | 300 | 2500 | 4y |
| Kawabata 2013[4] | 3(1M, 2F) | 20/10/19 | 140/81/72 | 50/15/27 | Halo-gravity traction | A+P | 64.3/81.5/62.5 | 625/675/412 | 2870/1413/2860 | 7y/3y/4y |
| Choksey 2015[13] | 1M | 13 | 46 | 26 | - | AO | 39.1 | - | - | 1y |
| Helenius 2016[3] | 22 | 11(1.7-19.9) | 70(12-105) | 21(-25-80) | Halo  traction, 9 | 9PO, 13AO+PO | 69 | - | - | 4.5(2-12)y |
| Gu 2019[14] | 7(5M, 2F) | 33.1(16-61) | 67.7(35-110) | 12.4(9-20) | Skull traction | AO | 83.1 | 145(120-180) | 180(100-300) | 2y |
| Wang 2019[7] | 10(8M, 2F) | 28.1(14-57) | 82.0(52-107) | 27.3(3-51) | Skull traction | AO+PO | 66.7 | - | - | 50.6m |
| Zhang 2021[19] | 26(11M, 15F) | 16(7-29) | 61.3±19.7 | 10.6±3.7 | Halo traction | PO | 82.7 | - | - | 43m |

**Supplemantary Table 1. Clinical results of in different literatures.**

| **Supplemantary Table 2. Comparison of our 12 cases and 80 cases in literatures** | | | | |
| --- | --- | --- | --- | --- |
|  | Our study | AO in literatures | PO in literatures | AO+PO in literatures |
| Numbers | 12 | 9 | 35 | 36 |
| Age | 24.67（14-41） | 28.07（8-61） | 15.95（5.2-29） | 19.89（6.1-57） |
| Gender |  |  |  |  |
| Male | 7 | 7 | 11 | 14 |
| Female | 5 | 2 | 15 | 9 |
| Preoperative LKA | 64.42（38-86） | 71.1（35-120） | 61.34（10-100） | 54.89（45-110） |
| Postoperative LKA | 16.83（-2-46） | 18.64（9-55） | 15.50（7-80） | 17.78（-25-58） |
| Correction rate(%) | 76.11 | 46.55 | 75.85 | 70.89 |
| Preoperative GKA | 35.5（10-81） | 39.4（20 - 68） | - | 54.9（15-107） |
| Postoperative GKA | 4.25（-22-39） | 6.9（-5-21） | - | 18.9（1-47） |
| Preoperative SVA | −4.8（-28-29） | −3.0（-29.1-6.6） | - | 5.8（-30-35） |
| Postoperative SVA | 9.0（-4.0-21） | 5.3（-17-16.5） | - | 9.3（5-20） |
| Preoperative T1 slope | −4.25（-24-19） | −27.9 (-66-14) | - | 12.3（-39- 20） |
| Postoperative T1 slope | 2.67（-19-14） | 4.1（-7-24） | - | 2.3（-14-25） |
| Preoperative JOA | 10.42（8-16） | 10.4（6-14） | 10.81（6-14） | 8.63（3.5-16） |
| Postoperative JOA | 15.25（11-18） | 15.4（14-17） | 13.01（14-17） | 10.52（2-17） |
| Preoperative NDI | 23.25（16-35） | 25.1（13-35） | - | 19.6 （10-27） |
| Postoperative NDI | 7.08（3-15） | 8.7（5-12） | - | 5.9（0-12） |
| Preoperative VAS | 6.33（4-8） | 7.0（4-9） | - | 7（5-9） |
| Postoperative VAS | 1.42（0-3） | 2.3（1-3） | - | 1.2（0-3） |
